# Eight weeks of supplementation with a multi-ingredient weight loss product enhances body composition, reduces hip and waist girth, and increases energy levels in overweight men and women

**DOI:** 10.1186/1550-2783-9-S1-P32

**Published:** 2012-11-19

**Authors:** Jennifer E Hofheins, Hector L Lopez, Scott M Habowski, Joseph P Weir, Tim N Ziegenfuss

**Affiliations:** 1 The Center for Applied Health Sciences, Stow, OH, USA; 2 Department of Physical Therapy, Des Moines University, Des Moines, IA, USA

## Background

While there are numerous natural products being marketed and sold that claim to help consumers lose body weight and body fat, very few undergo finished product-specific research demonstrating their safety and efficacy. Additionally, there is a growing interest in the role of adipokines in the development of Metabolic Syndrome and the regulation of body fat, blood pressure, insulin sensitivity, carbohydrate and lipid metabolism. The purpose of this study was to determine the safety and efficacy of a multi-ingredient supplement containing primarily raspberry ketone, caffeine, and Citrus aurantium (Prograde Metabolism™ [Metabo]) as an adjunct to an eight-week weight loss program.

## Methods

Using a randomized, placebo-controlled, double-blind design, 70 healthy men and women were matched for gender and body mass index and then randomly assigned to ingest 4 capsules per day of METABO or a placebo. Following baseline testing, both groups underwent eight weeks of daily supplementation, a calorie restricted diet, and supervised exercise training. All subjects were tested for changes in body composition via DEXA, serum adipokines (adiponectin, resistin, leptin, TNF-α, IL-6) and general markers of health (heart rate, blood pressure, and comprehensive clinical chemistry panels of sera and plasma) before and after 8-weeks of supplementation. Data were analyzed via ANCOVA using baseline scores as the covariate and statistical significance was set *a priori* at P≤0.05.

## Results

Significant differences were noted in: body weight (METABO: -2.0%; 94.3 ± 23.3 [wk 0] to 92.4 ± 23.4 kg [wk 8] vs. placebo: -0.5%; 91.0 ± 25.1 [wk 0] to 90.5 ± 24.9 kg [wk 8], P<0.01), fat mass (METABO: -7.8%; 37.2 ± 14.9 [wk 0] to 34.3 ± 14.8 kg [wk 8] vs. placebo: -2.8%; 32.6 ± 13.5 [wk 0] to 31.7 ± 12.7 kg [wk 8], P<0.001), lean mass (METABO: +3.4%; 52.8 ± 13.5 [wk 0] to 54.6 ± 13.8 kg [wk 8] vs. placebo: +0.8%; 50.5 ± 13.6 [wk 0] to 50.9 ± 13.6 kg [wk 8], P<0.03), waist girth (METABO: -2.0%; 104.1 ± 15.3 [wk 0] to 102.1 ± 14.7 cm [wk 8] vs. placebo: -0.2%; 104.6 ± 18.3 [wk 0] to 104.4 ± 18.1 cm [wk 8], P<0.0007), hip girth (METABO: -1.7%; 114.3 ± 13.4 [wk 0] to 112.5 ± 13.5 cm [wk 8] vs. placebo: -0.4%; 113.5 ± 15.1 [wk 0] to 113.3 ± 14.9 cm [wk 8], P<0.003), and energy levels (METABO: +29.3%; 3.0 ± 0.9 [wk 0] to 3.9 ± 0.6 [wk 8] vs. placebo: +5.1%; 3.3 ± 0.7 [wk 0] to 3.5 ± 0.9 [wk 8], P<0.04). We also observed effects/trends for maintaining elevated serum leptin (P<0.03), increased serum adiponectin (P<0.15), and decreased serum resistin (P<0.08) in the METABO group vs. placebo. No changes in systemic hemodynamics or clinical blood chemistries were noted between groups.

## Conclusions

These preliminary data indicate that METABO administration is a safe and effective adjunct to an eight-week diet and weight loss program. Ongoing studies are attempting to confirm these results and clarify the mechanisms by which METABO exerts the observed salutary effects.

## Competing interests

TZ and HL are consultants of Ultimate Wellness Systems Inc and have received direct remuneration for scientific and technical services related to dietary supplements.

